# Management of Major Limb Injuries

**DOI:** 10.1155/2014/640430

**Published:** 2014-01-05

**Authors:** Vijay Langer

**Affiliations:** Department of Reconstructive and Plastic Surgery, Army Hospital (Research and Referral), New Delhi 110010, India

## Abstract

Management of major limb injuries is a daunting challenge, especially as many of these patients have severe associated injuries. In trying to save life, often the limb is sacrificed. The existing guidelines on managing such trauma are often confusing. There is scope to lay down such protocols along with the need for urgent transfer of such patients to a multispecialty center equipped to salvage life and limb for maximizing outcome. This review article comprehensively deals with the issue of managing such major injuries.

## 1. Introduction

Major limb injuries involve many or all components of the limb architecture, namely, skin and soft tissue, osseous, vascular, and neural elements which makes for prompt and precise evaluation and management for optimizing functional outcome. In major limb trauma, often in the polytrauma setting, the burning question is whether to salvage or amputate the injured limb. Unfortunately, data on this issue is often conflicting and confusing with class I studies lacking. Even bodies like the Cochrane Collaboration are exceptionally silent on guidelines on the topic, especially as one set of guidelines considered correct in one part of the world may not necessarily be appropriate in another part. For both cultural and practical reasons, patients prefer to retain their own limb, even though deformed, provided it is painless and retains function [1]. This paper endeavours to analyse the various pros and cons of the subject and lay down protocols for clear cut management of the same. It also tries to answer various other associated questions which may translate into better functional outcomes for the patient with a major limb injury.

## 2. Things That Have Changed Since Malt: Advance or Atavism?

Ronald Malt performed the first replantation in 1962 at the Massachusetts General Hospital for a 12-year-old boy who amputated his right arm in a train accident [2]. Ever since then, innumerable replantations and revascularizations have been successfully carried out worldwide. However, the incidence of major upper limb injuries and amputated limbs that are replanted are on the decline throughout the developed world. This trend has been obvious since the early 1990s. Accurate statistics of the number of such injuries are difficult to obtain from different parts of the world, but finger and major replantations have been uncommon operations in USA [3, 4]. However, in the developing world, these injuries are increasing probably due to an increase in the number of motor vehicles without the corresponding development of infrastructure and the mushrooming of industries without the stringent adherence to safety precautions [4].

Despite innumerable successful limb salvage, macroreplants are still viewed with doubts and whether it is worth the risk involved. The reason is quite evident: experience of the units is dwindling. Thus, when an occasional such patient is seen, the easier option of amputation is resorted to. Rarely does a unit get the ideal case with a sharp cut injury. Mostly, these patients have an element of crush and avulsion. The golden adage in such challenging situations is: “just get in there and replant!”

Skin and bone are relatively resistant to the propagation of blast and fragment, but muscle offers little impediment, and contamination can track along fascial planes. The extent of contamination and devitalisation of tissue is often more extensive than initially apparent. In such cases, surgeons have traditionally performed guillotine amputations, transecting skin, muscle, and bone all at the same level. Although this is quick and requires little surgical skill, it makes closure difficult, and the final amputation level is often more proximal than necessary. Most surgical units now recommend fashioning definitive flaps at initial surgery, maintaining stump length and facilitating early closure [5].

## 3. The Injured Patient: A Holistic View

Clinical examination is of paramount importance. Such injuries are often associated with head injury, chest or abdominal trauma, and other significant musculoskeletal traumas. Detailed examination is necessary to exclude associated injuries. While assessing the patient, aggressive resuscitation should be initiated, preferably in the operation theatre annexe. Regional blocks should be given on arrival to alleviate pain and then patient taken up for surgery. Associated major trauma involving other systems should be tackled by another appropriate team of surgeons while the limb trauma is tackled by the reconstructive surgical team.

By definition, replantation is a reattachment of a body part that has been totally severed from the body (complete amputation). A revascularization is reconstruction of blood vessels that have been damaged in order to prevent an ischemic body part from becoming nonviable or necrotic (incomplete amputation). While for the latter, only one team will suffice, for the former, two teams are required; one prepares the amputated part and the other works on the recipient part.

## 4. The Decision: To Salvage or Amputate?

The shift from ligating an injured vessel to its primary repair resulted in corresponding fall in amputation rates from 50% in World War II to less than 2% in civilian injuries [6]. However patients reach the hospital late, thus necessitating contemplating amputation rather than limb salvage, especially during war. Operations are performed under suboptimal conditions, often by surgeons not trained in this field. The first successful hand replantation was reported by Chen et al. in 1963 [7]. Exhaustive work done at Kleinert Institute, Louisville, USA, has conclusively proven that salvaged upper limbs are functionally better than the best available prosthesis [8].

Indications and contraindications have been drawn up for replantations in Europe [9]. In general, the indications for replantation include amputation of a thumb, multiple digits, or a limb through the forearm, wrist, or palm. Amputations through the elbow and proximal arm should only be replanted if the part is a sharp amputation or has minimal crush injury. Single digits distal to the insertion of the flexor digitorum profundus tendon in the middle phalanx may be replanted in appropriate patients. A child with almost any body part amputated is a candidate, although the success rate of replantation in children is lower. In children, identification of vessels of operative size can be very challenging, not to mention their anastomosis [10, 11]. Absolute contraindications include severely crushed or mangled parts, amputations at multiple levels, prolonged warm ischemia more than 12 hours, and amputations in patients with other serious injuries or medical illnesses. Most units follow these guidelines. While these serve as a guide for practice in general, leading teaching units should continue to explore the limits of what is possible. This will allow redefinition of indications and contraindications for limb salvage at regular intervals, almost once every decade. The refining of indications is well worth the effort because a majority of patients are in the 20 to 30 years group and have a lot of productive life left [4].

Obtaining consistent good outcome for patients with complex injuries and providing a good quality replantation service challenge most health care systems. Introduction of the concepts of radical debridement, primary reconstruction, and early extensive rehabilitation combined with the advent of microvascular surgery and stable fixation devices has made the salvage of severely injured limbs a definite possibility.

Perhaps the most predictive indicator for success with replantation is the mechanism of injury. O'Brien has demonstrated significantly higher success rates with replantations of guillotine versus avulsion amputations [12]. Even as late as in the year 2003, it is widely believed by several bodies that it may be an unrealistic expectation to successfully replant severely crushed and mangled body parts [13]. The important questions to be answered are: can the limb be salvaged and if so, will the salvaged limb serve any purpose? Coupled with the high expectations of the patients and their families, it involves a major decision-making process. Even though limb salvage involves 2 to 7 secondary procedures and an average of 11 months for functional restoration [14–16], while this may be a problem in the West, in the developing world, it is not much of an issue because hospitalization is not as expensive.

The ischaemic time, especially of warm ischaemia, is one of the most important factors that influence the outcome. The longer the ischaemic time is, the greater the changes in cellular metabolism in the amputated segment are, especially in the muscles. These changes can produce permanent damage and a reperfusion syndrome after replantation. The order of the replant procedure is modified for major replantations of the hand and upper extremity. It is critical to minimize warm ischemia time to less than 4 hours to avoid muscle necrosis. Intravenous tubing or carotid shunts can be used to infuse and return blood to and from the amputated part.

When amputation is inevitable, performing early surgery enhances patient survival, reduces pain, disability, and shortens hospitalization [17]. The rate of secondary amputation for lower limb injuries undergoing limb salvage averages 25% within the first two years after seemingly successful limb salvage.

Limb salvage patients will have longer hospitalizations, more complications, and greater long-term disability. Data still support the aggressive limb salvage treatment for the younger patients, as total societal costs are less over the working lifetime of the individual [18]. Patients with multiple system involvement (Injury Severity Score >25) [17] often simply cannot withstand the persistent toxic load that a mangled extremity presents to them without exacting a toll on the overall system. Early amputation therefore is part of the life-saving process that must be considered even though the limb may be potentially salvageable.

The philosophy of saving life before limb cannot be emphasized enough when assessing the injured limb. The age of the patient and factors such as the site, the level and severity of crushed injury, the extent of soft tissue and bone loss, and contamination are all important in determining whether one should opt for an early amputation or persevere with limb preservation. A limb that is finally flail, painful, insensate, and nonfunctional will be inferior to amputation and prosthetic fitting. This is particularly true in the lower extremity where modern prosthetic appliances have proven to be effective in the restoration of almost normal function [19].

The initial examination is of utmost importance. Severe limb injuries must not distract the resuscitation team from the priorities of establishing an airway, optimising ventilation, and restoring circulatory volume, as limb injuries are rarely immediately life threatening, except those that cause exsanguination. If considered life threatening, amputation has been offered since times immemorial. But with aggressive modes of resuscitation in a large trauma centre, a well-co-ordinated multiple surgical team approach can indeed result in heroic limb salvage with saving of a life. Unfortunately, if a single general surgeon in a field hospital faces such a daunting situation where he cannot transfer the patient without probable risk to life, it may best be to amputate the traumatized limb and save life.

Of prime importance to limb survival is the competence of the vasculature distal to any injury. Local contusion, penetrating injuries, fractures, and, particularly, major joint dislocations may occlude or disrupt blood vessels. In the haemodynamically stable patient, examination of the distal pulses is crucial in assessing the peripheral circulation. A diminished or absent pulse strongly suggests a vascular injury and must be explained and managed promptly. Skin colour will also indicate tissue perfusion, and pallor or a blue-grey colour should arouse suspicion. Similarly, a low skin temperature indicates inadequate perfusion. A sensitive indicator is the capillary return—the normal prompt pink flush of the nail bed seen after transient compression. This response will be slowed or blue if the circulation is inadequate.

Peripheral nerves are very sensitive to ischemia, and sensation is lost early. Total insensibility in a hand or foot suggests ischemia as, except in patients with injuries to the brachial plexus or spinal cord, it is unlikely that all nerve trunks will have been damaged primarily in one limb. An inadequate distal circulation is never due to spasm in a traumatized limb. If distal ischemia is identified, more proximal pulses should be checked and any major deformity at the fracture site corrected and the splint device checked for local compression. Dislocations of major joints require urgent reduction. Doppler ultrasonography may be useful in evaluating limb perfusion, but if a vascular injury is suspected, arteriography provides the best definitive evaluation. Evidence of nerve injury may be difficult to obtain in the unconscious or multiply injured patient. Neurological function should be documented to allow later comparison.

In arterial injuries, successful results were obtained in arterial reconstruction procedures, which were held 6–8 hours after the event [20, 21]. Almost all of the amputations performed are late cases that were revascularized after 8 hours following the injury [22].

Replantation of a limb is complex and requires technically demanding surgery. Each step is critical. Ischemia of the main muscle mass of the amputated part can produce severe complications during and after surgery. Inadequate management may lead to failure, bleeding, and immediate death from the reperfusion syndrome. There may be poor function in the long term, although most patients are pleased to retain their limb. There is no clear objective measurement which can help the surgeon to make a proper decision and to predict the immediate and late results. Most articles simply describe the authors' experience [23–25]. Some have tried to use an algorithm for the treatment of traumatic amputation [26], but the measurements and assessment have been subjective and the numbers of patients small.

## 5. Who Should Do This Surgery?

When part of polytrauma, there is a risk that only life-threatening injuries are given attention and upper limb care is neglected. To carry on with limb salvage, after the life threatening risks are overcome calls for the resources of a major centre. Even if it is an isolated injury the extent of intensive monitoring needed, the possible requirement of considerable operating theatre time at short notice for secondary procedures make referral to high-volume centers advantageous. On the other hand, single finger and tip replantations do not need intensive postoperative care. Outcome mostly depends upon the hand and microsurgical skills of the surgeon. Digital replants will be more done in smaller centers by individual microsurgeons and they will post equally good results. Single most important variable is the availability and willingness of the trauma reconstructive surgeon willing to accept hardships and turbulent times. Salvage of these injuries depends upon the fortuitous availability of a combination of skilled manpower, appropriate decision making by the surgeon, and adequate infrastructure [4]. The unit should be on stand-by mode round the clock and there should be a sense of urgency in treating these patients.

## 6. Which Trauma Score to Follow

There is much confusion over which trauma score to choose in the limb trauma setting. Over the years, the Gustilo Mendoza Williams classification for assessment and prognostication of open limb injuries has been considered sacred. Apart from this, there have been many trauma scores which have been devised such as the mangled extremity severity score (MESS), the limb salvage index (LSI), the predictive salvage index (PSI), the nerve injury, ischemia, soft tissue injury, skeletal injury, shock and age of patient score (NISSSA), and the Hannover fracture scale-97 (HFS-97). All have been designed to assess a limb with combined orthopedic and vascular injuries. A mangled extremity severity score (MESS) has been devised to predict outcome after limb polytrauma, especially for the mangled limb (Table 1) [27]. Primary amputation is recommended if this score is high (≥7), or the limb is irreversibly ischaemic or gangrenous. Poor predictors of limb salvage are delayed revascularization (beyond 6–8 hours), presence of associated fractures, arterial ligation, and location of injury (popliteal). However, in some recent studies, it has been seen that successful limb salvage in patients with an MESS of ≥7 is possible with good functional outcomes [28–30]. MESS of ≥7 is not a good predictor for the need for amputation in patients especially with upper limb vascular injury, although an MESS of <7 remains a good predictor for patients who do not require amputation [30].

To add to the turmoil, a large multicentre study has documented poor sensitivity and specificity of these scores in type IIIB injuries where vascularity was intact [31]. Type IIIB open injuries of limbs are a major challenge in management being associated with a high incidence of nonunions [32], early and late infections [33, 34], a prolonged period of treatment [35], a high number of secondary procedures [36], poor functional outcome, and the possibility of secondary amputations [37, 38]. By definition, type IIIB can include a wide spectrum of injuries from the easily manageable to the rarely salvageable. Grouping all these injuries under the same code can lead to serious flaws in appropriate decision making, in prediction of outcome, or in comparing the results published from different series. As a result, there is a high degree of subjectivity and this has been the same conclusion of two major studies evaluating Gustilo's classification [39, 40]. It becomes necessary that there is a method available not only to predict salvage but also to provide guidelines in treatment and prognosticate the clinical outcome. In the absence of a vascular insult, the decision to amputate is mostly guided by the severity of damage to muscle units, with loss of bone. This is not specifically addressed by any of these scores. Observing the importance of the entity and paucity of any Indian study, the predictability of amputation or salvage in a mangled extremity by using the MESS in Indian patients was critically analysed. Though the MESS system was an excellent tool to predict primary amputation, it was found lacking in predicting successful limb salvage and final functional outcome [41]. To overcome these shortcomings, Ganga hospital open injury score was developed in 1994 to overcome the disadvantages of Gustilo's classification. After three clinical trials and suitable modifications, the score evolved to its present form (Table 2) [1]. This score has high sensitivity, specificity, and positive and negative predictive values. Another feature of the score was the high interobserver agreement, which was not experience dependent. The ability of the score to accurately predict salvage, even when the vascularity was not compromised, was a special advantage. This score, however, is advantageous for Gustilo type IIIA and IIIB injuries alone.

Further to the foregoing, based on an extensive literature search, no single trauma scoring system is superior to the other and none is the gold standard. Indeed, they may be considered guidelines to the final decision in management and functional outcome. Russell et al. [42] used limb salvage index and concluded that scoring assessments cannot replace clinical judgement. Poole et al. [43] commented that severity of injury of the soft tissues is closely associated with a high probability of amputation. Moniz et al. [44] related concomitant vascular and orthopaedic injuries as a good prediction tool for amputation. Durham et al. [45] reflected that scoring systems were able to identify the majority of patients who required amputation, but that prediction in individual patients was problematic and none of the scoring systems were able to predict functional outcome. Bosse et al. [46] questioned the very clinical utility of any of the scores. The results of a recent meta-analysis demonstrated that, in terms of physical outcomes, there is no statistically significant difference between amputation and reconstruction. On the other hand, assessment of psychological outcomes indicated that the reconstruction group has a better outcome. This meta-analysis, in essence, demonstrates that limb reconstruction in mangled lower limb injuries yields better psychological outcomes compared with amputation, without significant difference in physical morbidity [47]. The following may be prognostic indicators to poor outcomes following limb salvage, especially in a mangled limb: warm ischemia >6 hours, cold ischemia >12 hours, multilevel injury, extensive bone loss, nerve loss (especially tibial nerve), multiple joint disruption, advanced age, psychosocial disturbance, and rehabilitation compliance concerns. The decision for limb salvage versus amputation has to be finalized by the clinician judiciously after taking into consideration a combination of factors discussed.

## 7. How Is the Upper Limb Different from the Lower One?

Hand is used for function and foot is meant for mobility and bearing weight of the body. This alone is the difference between functional outcomes of limb salvage against amputations and use of prostheses. Function of the lower extremities primarily affects stance and ambulation, which current prostheses adequately support. Function of upper extremities, however, requires coordinated function of the digits and is dependent on sensation. Although current prostheses can provide gross motor movements such as grasp, in many instances they do not adequately restore fine motor function.

A lot of work has been done to substantiate superiority of limb salvage of the upper limb versus its amputation. Finger and indeed major replantations have had significantly better functional outcomes than prostheses [8]. Overall success rates for replantation approach 80%. In general, approximately 50% achieve two-point discrimination (2 PD) less than 10 mm [48, 49]. Several studies have determined the average replant to achieve 50% of normal function (i.e., 50% total active motion and 50% grip strength) [50, 51].

For a long time, surgeons have had the technical ability to salvage most, if not all, tibial fractures with vascular compromise. However, this is often “technique over reason” and often the end result is a physically, psychologically, financially, and socially crippled patient with a useless salvaged limb. The severe open tibial diaphyseal fracture remains a major treatment challenge to achieve fracture union and soft-tissue coverage in patients whose limbs are salvaged as brought out above. The Lower Extremity Assessment Project (LEAP) was a multicenter study of severe lower extremity trauma in the US civilian population [52–55]. In this study, functional outcomes were assessed for 601 patients who underwent reconstruction or amputation following severe, limb-threatening lower extremity trauma. Although few significant differences in functional outcome were found for those undergoing amputation versus reconstruction, outcomes for both groups were poor on average. In a recently completed 7-year follow-up of the LEAP patients, the study group found that outcomes for these patients had not improved [56]. Important findings from the LEAP study include the following.

1. The study could not recommend an existing index for determining when to perform amputation versus limb salvage.
2. Severe muscle injury had a strong influence on salvage. In essence, what the remaining long-term function will be with the existing motor units.
3. Bone loss was not particularly relevant.
4. Core morbidities, particularly alcohol use, created problems in long-term limb salvage.

## 8. Paradigm Shift from Lower Limb Amputations to Limb Salvage

Despite limb salvage rates having improved from 58% for Gustilo IIIC injuries in 1984 [57] to 94% with the use of free tissue transfer five years later [58] and the LEAP study report as discussed above, many reports have suggested that functional outcome is often poorer after successful lower limb reconstruction than after treatment with early amputation and a good prosthesis [59, 60]. These indeed have been a stumbling block in the struggle of lower limb salvage over amputation. However, as discussed earlier, it is worthwhile going the extra mile to salvage the lower limb.

There are, however, two absolute contraindications for lower limb salvage: anatomical complete disruption of the posterior tibial nerve in adults and crush injuries with warm ischemia time of more than 6 hours [61, 62]. This is so because the function of the limb is determined by the functioning of both motor and sensory nerves. For the leg this would primarily be the posterior tibial nerve as it both renders protective sensation to the limb and motors the foot flexors. A delay in revascularization can lead to life-threatening reperfusion injury with its complications.

Functional outcome depends upon intact skeletal framework and ligamentous integrity which are important for pain-free mobility. Beyond mid-tarsal joints, extent of available skeleton does not matter. Good after-care is more important than the choice of flap used for lower limb salvage.

## 9. How to Handle the Injured Limb: Precare

All unnecessary handling of the injured part without splinting should be avoided. The exceptions to this rule are when either severe deformity or ischemia of the limb distal to the fracture threatens survival of the soft tissues; reduction is then indicated. This is achieved by gentle traction and restoration of the normal anatomical alignment. Perfusion of the distal limb must be checked after any manipulation. Prehospital care must avoid further soft tissue injury. Splints are mandatory before the victim is evacuated. Strapping to the opposite leg is useful in solitary lower limb injuries, and splints can be produced by bandaging blankets or pillows around the limb. Wounds should be covered with a clean dressing, preferably one that is sterile. External bleeding should be controlled by a compressive pad and elevation. Bleeding vessels in the stump should not be clamped. Rapid transfer to hospital is then required. When a surgeon or a physician is faced with a patient with vascular injury in forward location, they should only control the hemorrhage, and not attempt definitive vascular repair. If limb viability is suspect, they can use temporary indwelling shunts to maintain circulation [63, 64]. These can be improvised from ordinary intravenous tubing or feeding tube under field conditions.

The amputated part should be inserted in a plastic bag and placed on ice as cooling attenuates reperfusion injury [65, 66]. It should not be placed directly on ice because this can result in a frostbite injury to the tissue. The amputated part should not be immersed in water because this has been demonstrated to make digital vessel repair more difficult and less reliable [67, 68]. The recommended ischemia times for reliable success with replantation are 12 hours of warm and 24 hours of cold ischemia for digits, and 6 hours of warm and 12 hours of cold ischemia for major replants (parts containing muscle).

## 10. Debridement

Debridement is the key to success in the management of major limb injuries. The term débridement was derived from the French verb “débrider” as early as 1810; it was used to denote “the action of cutting certain parts which—like a bridle—constrict or strangulate the organs which they cover” [69]. The extent of surgery appropriate for limb wounds was discussed in detail at the Inter-Allied Surgical Conference in 1917. The consensus that was reached emphasized the need for excision of the skin margin, generous extension of the wound, exploration through all layers, and excision of damaged muscle.

The present day concept of debridement is the even more aggressive form called wound excision. This encompasses excision akin to oncologic clearance with a 2-3 mm margin around the contaminated and grit laden tissues. It entails removal of dead and contaminated tissue that, if left, would become a medium for infection. For limb wounds, a pneumatic tourniquet and magnification in the form of loupes should be always used to reduce blood loss, distinguish surviving from devitalized tissue, and also prevent inadvertent iatrogenic trauma to vital structures. At the end of the procedure, the wound should be washed with copious quantities of saline, preferably as pulsed lavage and then soft-tissue coverage planned in the form of suturing, skin grafts, or flaps.

Infection, which increases amputation rate after a successful revascularization, is directly proportional to inadequacy of debridement [70]. Breidenbach III [71] reported an infection rate of 6% if wounds are thoroughly debrided (less than 10,000 bacteria per gram of tissue) and subsequently covered by a free flap in emergency cases. The correct level to which necrotic tissue should be resected is based on two observations: the surrounding soft tissue must be adherent to live bone and live soft tissue and bone must bleed [72].

Though earlier reports suggested that skin flaps apparently vascular (noted by colour and bleeding) later became devascularised [73, 74], more recent experience shows that the skin edges that bleed show viability [1].

Adequate debridement should be performed within 12 hours of injury for the best results [1]. The appropriate principle is “when in doubt-excise” in dealing with muscles, fascia and bone and “when in doubt-conserve” in dealing with the skin. If skin is found to be nonviable during the relook and repeat débridement procedures, it can be partially excised. It is important that the excision of the skin be done without tourniquet and débridement of deeper tissues under tourniquet control. Second look for repeat debridement may be required sometimes. Soft-tissue coverage should be attempted then. The International Committee of the Red Cross recommends an interval of five days, but practice in the developed world now tends towards shorter periods of 48–72 hours. The only indication for return to theatre before this time has elapsed is sign of sepsis or an offensive smelling dressing.

## 11. Bony Injuries

Unsatisfactory bone healing will defer weight-bearing which has a bearing especially in the lower limb salvage. Therefore, it is a crucial aspect of the final result after limb salvage.

### 11.1. Internal Versus External Fixation

Though occasional reports suggest significant infection rates in open intramedullary nailing of open fractures [75], the scare of increased incidence of infection and explantation after internal fixation in the patient with major limb trauma appears ill founded in the face of current practice of aggressive debridement. Bhandari et al., in a meta-analysis, found that, compared with external fixation, the use of internal fixation by unreamed nails decreased the risk of reoperation, superficial infection, and mal-union in open tibial fractures [76, 77]. Complications such as nonunion [78], pin tract infections, and chronic osteomyelitis may be significantly high. Performing a flap with a temporary bone fixation has the disadvantage of protracted treatment schedules. Once the flap is performed, definitive skeletal fixations have to be postponed until the flap settles. It is now accepted that internal fixation, in the presence of a well-excised and immediately covered wound, does not increase the rate of infection [1]. This would suggest superiority of internal fixation over external means of skeletal stabilization. The trend is shifting toward staged internal and external locking plates for skeletal stabilization.

### 11.2. Bone Grafting

There has been much debate on the use and timing of bone grafting for gap in bones after debridement. Trabulsy et al. reported significantly improved bony union following bone grafting before 12 weeks than after that period [79]. The ideal time for bone grafting in a patient with a high-energy tibial shaft fracture is also an unsettled issue. Some recommend bone grafting 5–7 days after debridement, but others argue that early bone grafting may result in resorption of the graft and/or increase in the rate of infection The early addition of bone grafts in open injuries has previously been reported to be safe [80, 81]. As soon as soft-tissue envelope is closed and noninfected, bone grafting may be considered. Thakur and Patankar have demonstrated excellent results using a protocol of early bone grafting and fixator dynamisation with monolateral fixators [82]. Cortico-cancellous, iliac bone grafts, to fill such bone loss, can be added during the index procedure, provided immediate wound closure, or wound cover, is possible [1]. Immediate bone grafting in patients who require it not only obviates the need for a secondary surgical procedure, but also has the added advantage of reducing the dead space. Placement of the grafts must be done in such a way that it does not cause tension, either in the soft tissues or the skin. This is invariably not a problem in the fractures of the humerus and femur but has to be undertaken carefully in the forearm and the lower leg.

### 11.3. Managing the Bone Gap

In the upper limb, radical bone shortening can be done so as to achieve primary, tension-free repair of the vessels, especially the veins. In the lower limb, with less than 4 cm gap, the bone ends can be approximated, but a bigger gap up to 6 cm needs nonvascularized bone graft and a bigger defect needs bone transportation which results in better bone stock than vascularized bone graft such as microvascular fibular or iliac crest graft. With acute shortening and stabilization using the Ilizarov frame, there is less need for free and local flaps. There is a decrease in the operating time and donor-site morbidity (important for patients with multiple organ trauma). It also provides a good option for restoring defects in severe cases with combined bone and soft-tissue defects in the same session. Its implementation for short bone defects (<3 cm) gives acceptable aesthetic and functional results. Angulation of the segments and subsequent graduated correction of misalignment reduces the length of shortening needed in patients with severe soft-tissue loss by sparing the bone from unnecessary debridement. It also permits definitive treatment using an external fixator device, enabling the possibility of early functional loading. These aspects suggest adopting this method for functional limb salvage after extensive complex high-energy injuries [83]. Articular injuries require early repair/reconstruction as the inevitably associated periarticular muscle damage will predispose one to stiffness or deformity unless the joint can be mobilized, or placed in a functional position.

### 11.4. One Bone Forearm

First described by Hey-Groves [84], the concept of a single bone forearm is quite useful after bone shortening or loss after debridement in the upper limb. Usually, the proximal ulna is fixed to the distal radius with good functional results [85]. Angle of synostosis has been a matter of much debate. However, currently, fusion at 30° pronation is considered to give best functional results [86].

## 12. Vascular Injuries

Revascularization of the limb is critical to the success of limb salvage. Even though about 95% of the injured limbs are successfully salvaged by early surgical intervention and revascularization [87, 88], the limb salvage rate may be lower in war injuries [89]. As per the American College of Surgeons Committee on Trauma in 2005, vascular injuries should be treated within 6 hours of injury to maximize the success of limb salvage. It is usually addressed after skeletal stabilization to prevent injury to the vessels again during skeletal manipulation. However, in some cases, fracture stabilization may follow the vascular repair [90]. For limbs that present late and are deemed salvageable, immediate temporary vascular shunts should be established [91, 92]. This would help to reestablish the distal circulation of the injured limb and buy time for subsequent debridement, skeletal stabilization, and vascular repair.

Vascular injury is clinically diagnosed on the “hard signs” of arterial trauma, namely, pulsatile external bleeding, rapidly expanding haematoma, absent distal pulse, bruit over the artery, or an ischemic limb [93]. These have 92–95% sensitivity for injuries requiring intervention. The vast majority of patients exhibiting these require intervention with a positive predictive value of 95%. Absent pulse is not a sensitive prognostic sign, as up to 25% of patients with major vascular injuries requiring repair have normal pulses distal to the injury. The “soft signs” or a wound adjacent to a great vessel only suggest vascular trauma. Preoperative arteriography may be indicated in such patients, especially if hemodynamically stable [94, 95]. However, its role is controversial. The positive predictive value of soft signs indicting abnormal findings on an arteriogram is only 35%. The vast majority of these lesions do not require emergent repair and there are many reports not in favour of routine use of arteriography [96], especially in patients who sustain military trauma and present late. In most of the patients, evidence of vascular injury can be made out clinically and by noninvasive techniques. Though Doppler ultrasound and CT angiography show high sensitivity, specificity, and accuracy [97], they may not be of value and may delay management as they are observer dependent and need sophisticated equipment and highly trained personnel for use.

Multiply injured patients with reduced tissue perfusion and oxygenation are at a high risk of developing compartment syndromes and, in such patients, early fasciotomy should be the rule, especially after revascularization [98]. Contrary to general belief, patients with open fractures may develop acute compartment syndrome from 1.2% to 9.1% of cases [99, 100]. These benefit with fasciotomy.

While there is a role of embolectomy in management of the acute limb ischemia not caused by trauma, in vascular injury caused by major limb injury, embolectomy is not only not of much benefit but can be harmful as there is intimal damage which causes thrombus formation in such injuries.

## 13. Nerve Injuries

Long-term functional outcomes of salvaged limbs are determined mainly by the nerve recovery and the optimum function of musculotendinous units. Nerve injuries are not given much attention in major limb injuries. In the presence of nerve injury, it is important to determine whether this is direct injury to the nerve or if there is a neurapraxia, anatomic disruption, or ischemia. The presence or absence of plantar sensation is thought to be critical, but there is little data to determine the effect of absent plantar sensation in a group of patients with limb salvage. It is important to document neurological status and explore if the wound is close to a major nerve trunk. Nerve injuries which can be primarily sutured lead to good motor recovery. Crush injury of a major nerve often requires complex surgical procedures like cable grafting or secondary tendon transfer procedures and a prolonged rehabilitation period. If avulsed nerve ends cannot be coapted, distal ends should be placed subcutaneously out of the zone of trauma for easier access at secondary procedures. If concomitant vascular repair has been carried out, this also prevents inadvertent injury to the repair site at secondary exploration.

## 14. Musculotendinous Units

Devitalized muscle should be radically excised. Tendons should be preserved if possible. Tendons avulsed proximally need to have all the associated muscles excised. After skeletal shortening at replantation, especially in the upper limb injury, tension adjustment in the slightly overcorrected status is important for better function.

## 15. Soft-Tissue Coverage

### 15.1. Timing of Coverage

The protocol of primary closure in open injuries is controversial and merits discussion. The widely accepted standard of care in the management of open wounds is to leave the wound open after débridement and to delay the closure to a later date. This concept has been carried over from the experiences and results of wounds sustained in war settings and needs to be reevaluated in the present climate of advanced clinical care [101, 102], especially as this would have the disadvantages of the risk of drying and desiccation of the exposed bone, periosteum, and soft tissues. There may be a need for secondary debridement for further secondary loss of tissues, which will lead to the need for a flap. The chances of contamination from hospital organisms also increase. It may be advantageous to evaluate wounds at the end of the debridement and follow a protocol of primary closure if the wound can be closed without tension. Early wound coverage has been documented to be vital to successful limb salvage. Godina reported 0.75% failure rate of 532 free flaps when they were done within the first 72 hours after trauma. The failure rate of the free flaps was 21.5% when the procedure was carried out three days or more after the injury [72]. Cierny et al. reported similar favourable results when the free flaps were in place within the first week after trauma. The flap failure rate, deep infection rate, and nonunion rate of the fractures were lower in the group with early wound coverage [103]. Exposed vital structures, such as vascular grafts, mandate coverage immediately. Some advocate coverage at the time of presentation, before the wound has been heavily colonized with bacteria [73], others within 6 days [104]. Ideally, definitive coverage would be performed when the wound is clean, stable, and before it becomes colonized with pathogens such as pseudomonas aeruginosa. The timing of the flap may be immediate, or staged, depending on the status of the soft tissues and the zone of injury. A “Fix and Flap” protocol is advocated in low-energy injuries and where the zone of injury is not extensive [1]. Providing cover after 5 to 7 days, even if performed before 10 days, delays bony union [105, 106].

The pioneering work of Lister and Scheker in 1988 led to the introduction of the concept of emergency free flaps, which, by definition, are performed within 24 hours of injury [107]. They proved flap survival rates equal to, if not better than, elective free flaps. This translated into significant reduction in infection, secondary procedures, and hospitalization cost.

Although some studies very convincingly indicate that free-flap failure is lowest if performed in an early time frame (first 3–5 days), other studies [108, 109] could not clearly indicate this advantage. Debridement of all devitalized tissue and subsequent free-flap anastomosis outside the zone of injury seem more important for flap survival than the time when the free flap is performed. It thus seems that, for flap failure, the time for performing the free flap is less important than for ultimate outcome of bone consolidation [108].

### 15.2. Type of Coverage

Quite often, in major limb injuries, soft-tissue defects are large and require large flaps. In such situations in revascularised limbs, distant flaps are a necessity. In the upper limb, distant flaps can be used quite comfortably while similar defects in the lower limb necessitate microvascular flaps. Success rate of 95–99% is consistently achieved for such flaps in microsurgical centres across the world, thus making them attractive as options for coverage. For combined bone and soft-tissue defects, composite free flaps should preferably not be used as geometry of need of bone and skin cover do not usually coincide.

A muscle flap is preferred to a fasciocutaneous flap because it fills up dead spaces, the blood supply is better, it makes better surface contact with the bone, allowing greater revascularization and hence better bone healing [109, 110]. It is possible that the perforators supplying the local fasciocutaneous flaps could be injured during high-energy trauma despite which numerous studies have demonstrated success using local fasciocutaneous flaps [111, 112]. However, it is commonly agreed that, in general, flap failure rate is much lower for free flaps.

## 16. Management of the Mangled Extremity

The mangled extremity presents a daunting challenge to a trauma surgical unit. A mangled extremity is defined as high-energy transfer or crush resulting in a limb with injury to three of four systems in the extremity. The management of such severe and complex injuries can be a daunting task. The decision to undertake limb salvage or to amputate is to be taken urgently. Criteria for immediate amputation are shredded muscle and nerves beyond elbow or knee, especially posterior tibial nerve in the lower limb, crushed or mangled limb with more than 6 hours of arterial occlusion upon arrival, associated mangling or severe injury to ipsilateral foot or hand, severe associated polytrauma with associated persistent hypothermia, acidosis, or coagulopathy. If the decision to opt for limb salvage is taken, the patient and the surgical team should be ready for 5 to 7 operative procedures in totality. 30–50% of the limbs only are usefully employed if salvage is successful and eventual amputation occurs for 30% of Gustilo IIIC fractures at presentation (discussion on the American College of Surgeons Committee on Trauma 2002). 17% of such patients have concomitant life-threatening injuries that have to be energetically managed [113]. However, limb salvage rates in these injuries have improved greatly primarily because of new techniques in soft-tissue reconstruction. Toe movement and a sensate sole are strong indicators for successful functional restoration following lower limb salvage.

The decision to salvage or amputate the mangled limb has generated much controversy in the literature, with studies to support advantages of each approach. Various scoring systems have proved unreliable in predicting the need for amputation or salvage; however, a recurring theme in the literature is that the key to limb viability seems to be the severity of the soft-tissue injury. Factors such as associated injuries, patient age, and comorbidities (such as diabetes) also should be considered. Attempted limb salvage should be considered only if a patient is hemodynamically stable enough to tolerate the necessary surgical procedures and blood loss associated with limb salvage. For persistently hemodynamically unstable patients and those in extremis, life comes before limb. The Lower Extremity Assessment Project (LEAP) study attempted to answer the question of whether amputation or limb salvage achieves a better outcome. The study also evaluated other factors, including return-to-work status, impact of the level and bilaterality of the amputation, and economic cost. There appears to be no significant difference in return to work, functional outcomes, or the cost of treatment (including the prosthesis) between the two groups. A team approach with different specialties, including orthopaedics, plastic surgery, vascular surgery, and trauma general surgery, is recommended for treating patients with a mangled extremity, with an individualized, rational, and realistic informed decision taken to either salvage the limb or amputate it [114].

A special mention needs to be made of the management of the mangled limb in the pediatric population. Due to specific anatomic and physiological characteristics, there are great differences in diagnostics and treatment. Children who received definitive treatment at a paediatric trauma centre had between 3 and 6 times higher odds of having a survival advantage than if treated at an adult trauma centre [115]. Even as an MESS of 7 or greater is a predictor of amputation in adults by many, pediatric patients with a high risk of amputation can be identified by using MESS with threshold of 6.5 [116]. It has also been reported that children with a MESS ≥ 7 underwent primary amputation less frequently than adults. However, a recent study quoted that while injury severity scores have been developed to aid decision making in adults, evaluation of their use in children is limited. All of the scoring systems had poor specificity and would have recommended amputation in several limbs that were successfully reconstructed. Currently available injury severity scores behave differently in children and adults. In their current format, these scores should not be used as an absolute indication for early amputation in children [117].

## 17. The Future

The future demands the society to make salvage surgical procedures for mangled and amputated extremities available to many more potential beneficiaries. In developing countries, this will involve education and sensitization of medical and paramedical professionals, increasing public awareness, building up transport systems and infrastructure, and training skilled manpower. Also on the frontier is the burning topic of hand transplantation. First done in 1998 [118], more than a dozen have been done thus far. Proponents of the technique claim early survival rates of hand allografts that have exceeded the initial success rates of any organ previously transplanted [119]. They are considered to have better functional outcome than prostheses and comparable results to replants done at the forearm level [8]. Despite these reports, hand surgery community seems to be largely skeptic toward the topic, mainly due to ethical issues, risk-benefit balance, and life-long host immunosuppression and its inherent complications. At the time of writing this paper, there does not seem to be any activity toward lower limb transplantation, primarily because the current prostheses give good functional outcomes.

## 18. Conclusion

Major limb injuries have attracted a lot of controversies over the past decades. They are injuries that result in much morbidity and functional deficit if not managed energetically. An urgent intervention and restoration of lost tissue or cover to optimize functional outcome is the key to success. The decision to salvage limb versus amputation in the patient with major limb injuries should be individualized due to many factors involved. The existing guidelines on the topic are blurred and confusing. This paper endeavors to crystallize decision making in such devastating injuries.

## Figures and Tables

**Table 1:** Mangled extremity severity score.

Skeletal/Soft tissue group		
Low energy	Stab wounds, simple closed fractures, small caliber gunshot wounds	1
Medium energy	Open or multiple level fracture, dislocations, moderate crush injuries	2
High energy	Shotgun blast (close range), high velocity gunshot wounds	3
Massive crush	Logging, rail road, oil rig accidents	4

Shock group		
Normotensive hemodynamics	Blood pressure stable in field and operating room	0
Transiently hypotensive	Blood pressure unstable in field but responsive to intravenous fluids	1
Prolonged hypotensive	Systolic blood pressure <90 mm Hg in field and responsive to intravenous fluids only in operating room	2

Ischemia group		
None	Pulsatile limb without signs of ischemia	0
Mild	Diminished pulses without signs of ischemia	1
Moderate	No pulse by doppler, sluggish capillary refill, paresthesia, diminished motor activity	2
Advanced	Pulseless, cool, paralysed and numb without capillary refill	3

Age group		
<30 years		0
30–50 years		1
>50 years		2

If ischemia time more than six hours, add 2 points.

**Table 2:** Ganga hospital open injury severity score.

Covering structures: Skin and fascia	Score
Wounds without skin loss	
Not over the fracture	1
Exposing the fracture	2
Wounds with skin loss	
Not over the fracture	3
Over the fracture	4
Circumferential wound with skin loss	5
Skeletal structures: Bone and joints	
Transverse/oblique fracture/butterfly fragment < 50% circumference	1
Large butterfly fragment > 50% circumference	2
Comminution/segmental fractures without bone loss	3
Bone loss < 4 cm	4
Bone loss > 4 cm	5
Functional tissues: musculotendinous (MT) and nerve units	
Partial injury to MT unit	1
Complete but repairable injury to MT units	2
Irreparable injury to MT units/partial loss of a compartment/complete injury to posterior tibial nerve	3
Loss of one compartment of MT units	4
Loss of two or more compartments/subtotal amputation	5
Comorbid conditions: add 2 points for each condition present	
(1) Injury-debridement interval > 12 hrs	
(2) Sewage or organic contamination/farmyard injuries	
(3) Age > 65 yrs	
(4) Drug-dependent diabetes mellitus/cardio respiratory diseases leading to increased anesthetic risk	
(5) Polytrauma involving chest or abdomen with ISS > 25/Fat embolism	
(6) Hypotension with systolic blood pressure < 90 mm Hg at presentation	
(7) Another major injury to the same limb/compartment syndrome	
